# Expectations of Care, Perceived Safety, and Anxiety following Acute Behavioural Disturbance in the Emergency Department

**DOI:** 10.1155/2011/165738

**Published:** 2011-07-10

**Authors:** Magdalen Lim, Tracey Weiland, Marie Gerdtz, Andrew Dent

**Affiliations:** ^1^St. Vincent's Hospital Melbourne and The University of Melbourne, 41 Victoria Parade, Fitzroy, VIC 3065, Australia; ^2^School of Nursing, Melbourne School of Health Sciences, The University of Melbourne, Melbourne, VIC 3010, Australia; ^3^St. Vincent's Hospital Melbourne, 41 Victoria Parade, Fitzroy, VIC 3065, Australia

## Abstract

*Objective*. We explored perspectives of emergency department users (patients and visitors) regarding the management of acute behavioural disturbances in the emergency department and whether these disturbances influenced their levels of anxiety. *Methods*. Emergency department patients and visitors were surveyed using the State-Trait Anxiety Inventory, and a purpose-designed questionnaire and semistructured interview. The main outcome measures were themes that emerged from the questionnaires, the interviews, and scores from the state component of the State-Trait Anxiety Inventory. *Results*. 70 participants were recruited. Users of the emergency department preferred behaviourally disturbed people be managed in a separate area from the general emergency department population so that the disturbance was inaudible (*n* = 32) and out of view (*n* = 40). The state anxiety levels of those that witnessed an acute behavioural disturbance were within the normal range and did not differ to that of ED patients that were not present during such a disturbance (median, control = 37, Code Grey = 33). *Conclusions*. Behavioural disturbances in the emergency department do not provoke anxiety in other users. However, there is a preference that such disturbances be managed out of visual and audible range. Innovative design features may be required to achieve this.

## 1. Introduction

Violence in the health sector and in particular, emergency departments (EDs) is well documented and spans decades [1–3]. The Australasian College for Emergency Medicine states that “acts of violence” include “verbal abuse, threats, and aggressive behaviours, in addition to acts of physical-contact violence” [4]. Violence in the emergency department is arguably an inevitable consequence of an ED case mix-those with conditions that are life-threatening, produce unbearable pain or altered mental states are at risk of agitation which can escalate to violence.

 Patients are the main users of healthcare systems. However, previous studies conducted in the emergency department have focused on staff perspectives [5–7] and the profiles of perpetrators of violence and aggression [5, 7–11]. To our knowledge no research has examined the perspectives of users present in the emergency department during violent or potentially violent incidents. Eliciting users' opinions can contribute to the quality of healthcare and highlight possible flaws or strategies that are not apparent to healthcare professionals or administrators. Additionally, the impact of acute behavioural disturbance on the anxiety levels of ED users remains unexplored. 

We developed a questionnaire based on a 5-point Likert Scale and a semistructured interview. Using these tools and the State-Trait Anxiety Inventory, we surveyed patients and visitors to the emergency department. Our primary objective was to explore users' expectations of care regarding violence, and their perceptions of safety and environmental influences in these episodes. Our secondary objective was to compare the levels of anxiety between ED users who were exposed to an acute behavioural disturbance and those who were not.

## 2. Materials and Methods

### 2.1. Study Design

This was a prospective, cross-sectional study of two groups of users (patients and visitors to the emergency department). The convenience sample of participants included those who had witnessed the occurrence of an acute behavioural disturbance (by other patients) during their stay in the emergency department, and a convenience sample of those who did not.

### 2.2. Setting

This study was conducted in the emergency department of St. Vincent's Hospital Melbourne (STV), a metropolitan public teaching hospital in the state of Victoria, Australia, between 1 December 2007 and 31 April 2008. The hospital has a coordinated clinical and security-based procedure to respond to episodes of actual or potential user violence. This response is known as a Code Grey (CG) and involves an announcement and the presence of a team comprising healthcare staff and security officers managing the aggressive user. In addition, a behavioural assessment room (BAR) was specifically created by the hospital in 2003 to manage a patient whose condition causes behavioural symptoms that endanger themselves or others. This room provides a safe environment for staff, the person experiencing an acute behavioural disturbance, and other ED users. The BAR is a small room that is located external to the ED, next to the ambulance bay and the triage area. It functions as a short-term low stimulus environment for assessing and managing acutely agitated patients. Once patients who enter the BAR are assessed, and treatment for their agitation is commenced, care will continue in the emergency department.

### 2.3. Selection of Participants

Two groups of ED users were recruited: (1) a group that witnessed an acute behavioural disturbance and (2) a control group that did not. For the first group, patients or visitors were eligible if they were present in the emergency department during at least one episode of acute behaviour disturbance. Patients or visitors were eligible for the control group if at the time of recruitment they were present in the emergency department and an acute behavioural disturbance had not occurred in the previous four hours. Users were excluded from participation if they were unable to communicate in spoken or written English, were under the age of eighteen, prisoners, or unable to provide consent.

### 2.4. Methods of Measurement

Three instruments were used: (1) a questionnaire comprising of ten statements based on a 5-point Likert Scale, (2) a semistructured interview, and (3) the State-Trait Anxiety Inventory (STAI).

A purpose designed questionnaire and semistructured interview were developed by an emergency physician, a psychologist/researcher, a nurse academic with ED experience, and a medical student using a Delphi panel process involving iterative feedback. This resulted in an interview schedule comprising one open-ended and four closed questions, and ten statements that could be rated according to a five-point Likert Scale.

 Specific questions such as “*Were you aware that violent patients present to the ED?*” and “*If a patient experienced an acute behavioural disturbance in a cubicle near you, how would you like staff to manage the CG with relation to yourself?*” were asked to prompt the participants if they were unable to give a response to the open-ended question. Other questions in the interviews for the participants who witnessed an acute behavioural disturbance included “*Did you hear the Code Grey announcement?*”, “*Do you know what a Code Grey is?*”, “*Did you observe any violence or physical interactions around another patient today while in the ED?*”, and “*Did you hear any loud/inappropriate language today while in the ED?*” For the control group, a variation of the same questions posed in a hypothetical situation was asked. Explicit definitions of the terms “violence” and “aggression” were not provided but were left open for the participants' interpretation.

The State-Trait Anxiety Inventory is a self-reported instrument containing 40 statements divided equally between measuring state anxiety (anxiety experienced at that moment) and measuring trait anxiety (a person's baseline anxiety level that is related to his or her personality) [12, 13]. Scores for the state and trait components each range from 20 to 80 with a higher score corresponding to higher anxiety levels. Typical scores for people with anxiety range from 47 to 61. This scale is the most widely validated scale of anxiety [14].

### 2.5. Data Collection and Processing

Previous research demonstrated that the highest frequency of patient aggression was between midnight and 4 am [15] although it could occur any time in a 24-hour period. To facilitate recruitment, the recruiting researcher was informed of all CGs via a paging system with the exact timing and location of the CG. Eligible patients or visitors were approached and a brief description of the study was given. Participants were provided with a definition of a CG to ensure informed consent. 

A plain language statement which explained the nature of the study was given to eligible participants. Verbal consent was recorded and the semistructured interview was conducted at the patients' bedside or in a private area for visitors. Interviews were recorded with a digital tape recorder to ensure accuracy of transcriptions and minimise distraction to the participants that may occur with note taking.

Following the interview, the STAI was administered. Depending on the physical condition of the participant, this was answered in either written format by the participants or administered verbally by the researcher who then recorded participants' responses. Demographic information of patient participants was retrieved from the electronic patient administrative system. Demographic information was obtained directly from the participant and recorded in written form if the participant was an ED visitor.

A similar procedure was followed for the control group.

Information about the CGs was retrieved from Riskman, a database used to document information about the causes, management, and locations of the aggressive patients and their demographics.

This study was approved by the St. Vincent's Hospital Human Research Ethics Committee.

### 2.6. Outcome Measures

Data collected were analysed for emerging themes from both the questionnaires and semistructured interviews. The scores from the STAI were tallied.

### 2.7. Sample Size

Since other outcome measures were either qualitative or exploratory in nature, our sample size calculations were based on the quantitative outcome measure, the state component of the STAI. We estimated, using Sample Power, that a 5% difference between means would require a sample size of 34 in each group assuming a variance in means of 13% and a common standard deviation of 10% with power set at 90%. For this reason we sought a sample size of 70 (35 per group).

### 2.8. Primary Data Analysis

Data were collated using Microsoft Excel 2003 and analysed using SPSS for Windows (Version 15). For interview items answerable using a Likert scale, responses were collapsed into either: strongly disagree/disagree/neutral or agree/strongly agree. Descriptive statistics (number, percentage, 95% confidence interval) were calculated for responses to all closed questions and questions answerable using a Likert scale. Comparisons between the two groups of participants were made using Fisher's exact test for 2 × 2 contingency tables. Data for anxiety were analysed using independent samples *t*-test after assessing assumptions. Additional univariate analyses of covariance were conducted for state anxiety to control for the effects of presenting complaint (illness/injury), participant type (patient/visitor), and, among patients only, acuity as rated on the Australasian Triage Scale. For all inferential statistical calculations, two-tailed tests of significance were used and alpha was set at 0.05. The remaining quantitative data were analysed using descriptive statistics only (mean, the 95% confidence interval for the mean, the median, the interquartile ranges, numbers, and percentages).

Responses to open-ended interview questions were subject to thematic analysis using the Framework Method [16]. All responses were reviewed by one researcher who identified themes. Two independent raters then categorised all responses into the set themes. Interrater reliability was conducted using Cohen's Kappa to determine consistency in rating and discrepancies were resolved through discussion and mutual agreement. Data saturation was achieved when 15 participants had been recruited.

## 3. Results

A total of 265 CGs occurred during the study period of 3 December 2007 to 13 March 2008. A total of 100 patients were approached for participation in the study. Ten participants were excluded because of inadequate written or spoken English proficiency, or inability to provide informed consent. Thirty-five users were recruited to the control group, and 37 users were recruited to the group who witnessed an acute behavioural disturbance. Of the latter, two participants failed to complete the STAI due to medical procedures being done. 

**Figure 1:**
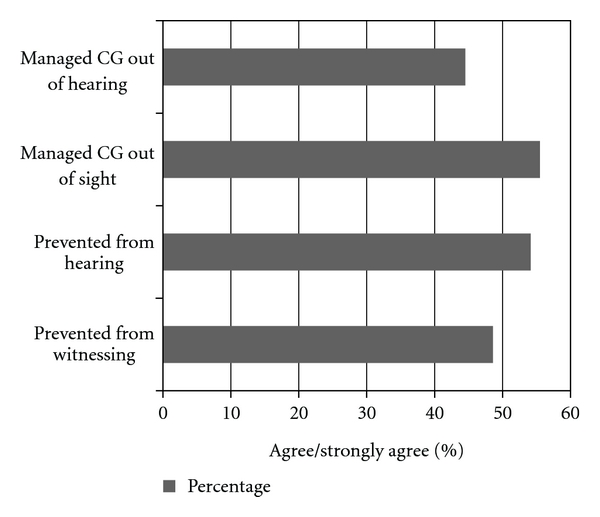
Overall percentage of participants who agree that the CGs should be managed and prevented out of their sight and hearing.

**Figure 2:**
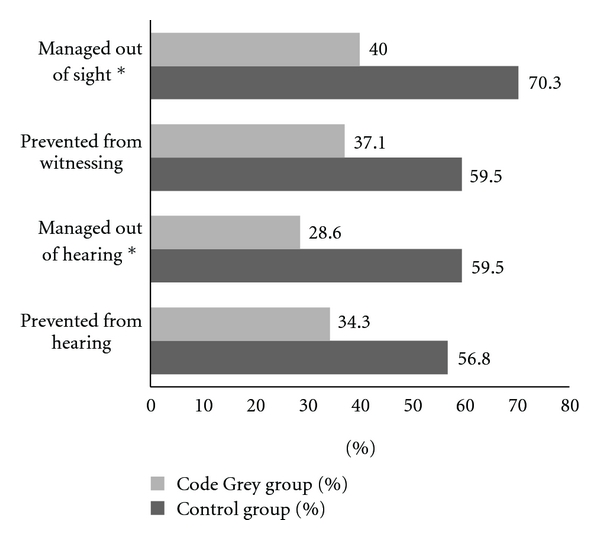
Comparison in the attitudes between participants who witnessed a Code Grey (CG) and control participants. *Denotes significant group difference, *P* < 0.5.

 The mean age of the participants was 48.1 years (95% CI: 44.3–51.9; range: 20–77 years), and was comparable across groups (*t *(70) = 0.63, *P* = 0.53). The gender distribution was even (males, females *n* = 36) across the sample and similar between groups (Code Grey group, 20/37 female; controls, 16/35 female; *P* = 0.56). Of 72 participants interviewed, nine were visitors and 63 were patients. Of the 63 patients, 17 presented with injuries due to accidents, and 46 presented with illnesses. Approximately half the number of participants knew what a CG was (52.8%, *n* = 38). When asked about their experience of having a CG occurring during their ED attendance most participants in the Code Grey group reported hearing the announcement (64.9%; *n* = 24), approximately one-third (12/37) heard inappropriate language, and few observed physical interactions (2.7%; *n* = 1).  Table 1 summarizes the perspectives of participants regarding witnessing a Code Grey, and Table 2 summarizes the perspectives of the control group.  Figure 1 reveals the percentage of users who preferred the aggressive patients be managed away from them.  Figure 2 shows the difference in preferred management between the group that witnessed an acute behavioural disturbance and the group that did not.

Thematic analysis revealed three dominant themes from the semistructured interviews. These themes can be broadly classified into acceptance (33.3%), separate management (38.9%), and those who refrained from providing comments (27.8%). One responder who felt that such violence was part of the system said, “*There are people some with alcohol, some with psychiatric problems. That's what you get when you come into an emergency department in a hospital.*” Another participant admitted that he did not feel affected by the acute behavioural disturbance probably because he was a male and could better defend himself if need be. Several respondents stated their preference that patients should be screened before allowing entrance into the emergency department, but concurred that it was not a guaranteed method of preventing ED violence. Participants also revealed that they either wanted the agitated patient removed from the emergency department if the patient could not be adequately calmed down or to change their own locations. A few suggested creating a separate section to cater to this particular population of patients. Participants also believed that patients should be carefully screened before entry into the emergency department. Responders also advocated that they wanted to be informed of what was going on if there was an acute behavioural disturbance occurring in the emergency department. They would like to hear simple reassurances from healthcare staff to ease their anxiety after the commotion. Most participants, however, acknowledged that healthcare staff were doing their best to manage the situation and felt grateful for their effort.

**Figure 3:**
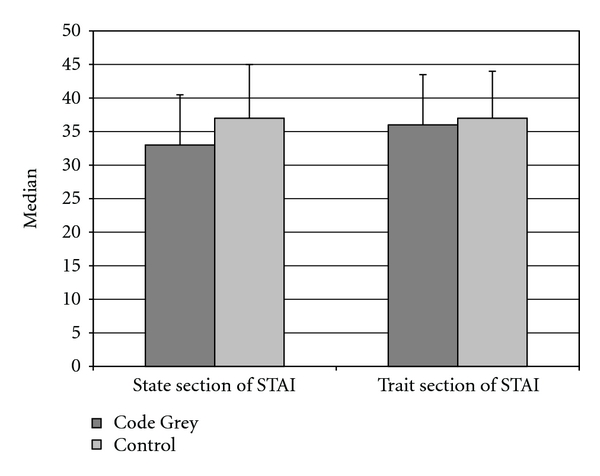
Median (IQR) State-Trait Anxiety Inventory state and trait anxiety scores of participants according to Code Grey exposure.

**Figure 4:**
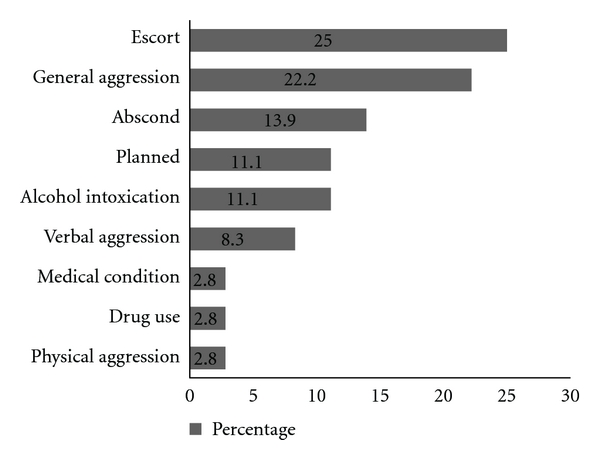
Distribution of Code Grey events by contributing factors as documented by security staff.

**Figure 5:**
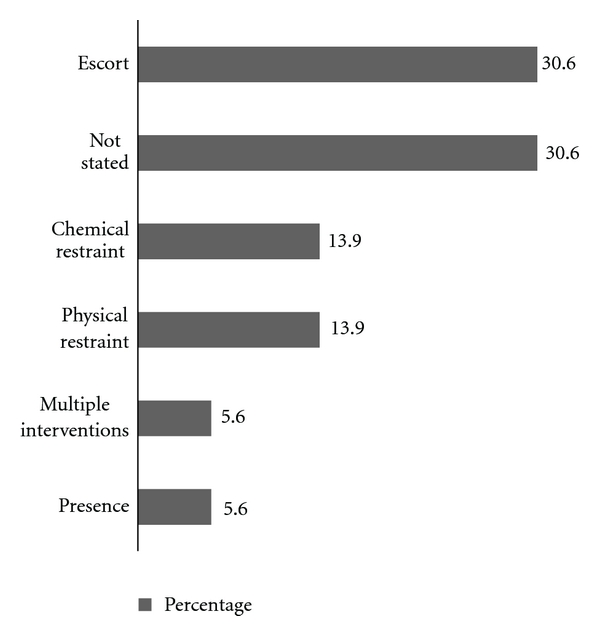
Distribution of Code Grey events by management strategies used.

Figure 3 demonstrates the results of the State-Trait Anxiety Inventory. There was no significant difference in state anxiety between those present or absent during a Code Grey (*F *(1, 69) = 2.002, *P* = 0.162). This outcome was unaffected by controlling for covariates presenting complaint (illness, injury) and participant type (patient, visitor; *F* (1, 66) = 3.35, *P* = 0.072). Similarly, amongst patients, the acuity of their condition (Australasian Triage scale, 1–5) was not a significant mediator of the outcome (*F* (1, 59) = 1.95, *P* = 0.168). Information regarding 36 CGs was retrieved from Riskman. Seventy-two percent (26/36) were male and 56% (20/36) were managed in the BAR.  Figure 4 indicates the causes of the CGs and Figure 5 demonstrates the resulting management involved.

## 4. Discussion

Acute behavioural disturbance that cannot be diffused through discussion with the user is typically managed using physical, chemical, or mechanical restraints, or in a limited number of EDs, by transferring the patient into a BAR. Security officers are employed by some hospitals as part of their security system [17]. In our hospital, security officers are present on site twenty-four hours a day. In other institutions, police are employed to provide extra security in the ED [18, 19]. One study suggested that installing security equipment in the hospital might unnecessarily frighten ED users and discourage them from coming into the emergency department for treatment [17]. Most respondents in our study, however, expressed satisfaction when the perpetrators were removed from the emergency department and taken to the BAR to be managed. This indicates that future designs of emergency departments should strongly consider allocating a separate safe space that is specialized for managing violent patients. For existing emergency departments, certain beds in a particular section can be maintained for the sole management of aggressive patients, similar to the concept of a separate resuscitation bay in the emergency department. This segregation can achieve two objectives: minimize the effects of aggression on the other users and provide privacy and dignity for the acutely disturbed patients.

Several participants in our study also suggested that healthcare staff screen patients for potential violence before allowing them entry into the emergency department. This is not as straightforward as it looks. There is a variety of reasons why patients may turn violent and many triggers are not immediately apparent. Lidtz and colleagues also demonstrated that it was very difficult to accurately predict if someone was going to be violent [20], although another study demonstrated that it was possible to pick up on signs of impending violence [21]. Therefore, being vigilant and transporting the patient that is exhibiting signs of aggression to a separate space for safe management may be optimal. Providing aggression management training may augment this process [22, 23]. However, there are situations when it is impossible to separate an aggressive patient. In such cases only expedited and meticulous management would suffice. One participant likened the scenario to that of one she encountered in her hospitality profession—“*manage the situation as quickly as possible to reduce collateral damage.*”

Lastly, responders also recommended that they would like to be informed of what is happening and whether the disturbance is under control. They would feel more reassured to know that the situation is under control. Therefore, it is important to note that as much as we would like to shield the other users from the effects of acute behavioural disturbances, sometimes it is inevitable to do so, and as healthcare professionals, we should try to alleviate their potential fears and anxieties by being honest and upfront with them.

We observed a similar level of state anxiety among those present during a Code Grey and participants that were not present during the department during a Code Grey. This outcome was unaffected by presenting complaint, patients type and acuity (patients only). Mean normal state anxiety scores have previously been reported as 35.7 for men and 35.2 for women [24]. In the present study mean total state anxiety was 34.3 (95% CI 31.9–36.9) suggesting a level of anxiety in this population that approximates what would be expected among the normal population. This is consistent with other studies in the ED setting [25].

## 5. Limitations

This study interviewed users from a single emergency department and may, therefore, not be a representative sample of all emergency departments, particularly those that do not have a BAR in place or that lack policies for managing acute behavioural disturbances. Code Greys are unpredictable and can occur anytime during a twenty-four hour period [10, 26–28]. Although the recruiting researcher responded to CGs across a 24-hour period, the possibility of selection bias cannot be excluded as the recruitment was undertaken by a sole researcher who was unable to attend to all CGs occurring at odd hours.

 The study may also have been limited by participation bias. Many participants that declined to be interviewed were older patients who were too weak from their conditions. It might be possible that they had different viewpoints from those who were willing to participate in the study. Moreover, participants who could not take part in this study due to a language barrier might also have differing opinions. Language barriers could make it difficult for patients to understand what was happening. They might also be less reassured by staff due to the language difficulties. Lastly, there were patients who required contact precautions due to the nature of their medical conditions. This made it logistically difficult to record the interview or for the patients to answer the questionnaires. 

 Results may differ if more visitors are interviewed since visitors are generally more alert than patients, and more aware of their surroundings and of any CGs that occur. A comparison between emergency departments with and without a specialized space to manage aggressive patients may also provide insight into the possible benefits of designing emergency departments with a separate space to manage such patients. Lastly, interviews conducted with patients who are violent may assist in elucidating patterns of violence within the emergency department.

## 6. Implications

Our study had shown that there was generally a high tolerance for perpetrators of violence, and most users are appreciative of the efforts of healthcare staff. As emergency nurses tend to be the ones that spend the most time with patients, they would benefit from courses that impart skills of observing impending aggression so that these patients can be safely moved to a separate space for management that benefits all ED users and staff. Emergency nurses should also be aware that in circumstances where it proved unrealistic to shield other users from the effects of acute behavioural disturbances, providing information to users could alleviate their anxieties and provide reassurance. These minor changes in current strategies should not be overlooked as they contribute to the overall quality of healthcare, for both staff and ED users.

## 7. Conclusion

In conclusion, this study provided greater depth in the current literature regarding the management of aggression in an emergency department setting. To the authors' knowledge, none of the studies in the current literature had included the perspectives of ED users and none have sought to assess the impact of CGs on patient/visitor anxiety. Since patients and visitors constitute the bulk of ED's users, it is crucial to determine what their perspectives are when violence occurs in their presence.

##  Conflict of Interests

The authors report no conflict of interests.

##  Authors' Contribution 

All authors participated in the design of the study; M. Lim collected data and drafted the manuscript; T. Weiland and M. Lim analysed data; all authors participated in the interpretation of results; T. Weiland and M. Gerdtz edited the final paper; all authors approved the final paper.

## Figures and Tables

**Table 1:** CG participants' attitudes regarding Code Greys.

Statement	Agree (%)	Disagree/neutral (%)
You feel threatened by what you have seen.	2.7	97.3
		
You feel threatened by what you have heard.	5.4	94.6
You were afraid of being harmed by the Code Grey event.	2.7	97.3
		
After observing this Code Grey, it will affect your decision to return to this emergency department in the future for treatment.	100	0

	Important (%)	Not important/neutral (%)

The privacy of the person experiencing the Code Grey was respected.	78.4	21.6
The dignity of the person experiencing the Code Grey was respected.	75.7	24.3

**Table 2:** Control participants' responses to statements in the questionnaire. The responses here were provided based on hypothetical situations proposed to the participants.

Statement	Don't know	Yes	No
If you were in the emergency department while there was a Code Grey event as I have just described, do you think you might feel threatened by what you may see or hear?	4	8	23
Do you think you might be afraid of ever being harmed during a Code Grey event?	7	12	16
Now that you know what a Code Grey event is, will it affect your decision to return to SVHM for treatment?	1	2	32
